# Prevalence and Characteristics of Knee Osteoarthritis Among the General Public in Saudi Arabia

**DOI:** 10.7759/cureus.47666

**Published:** 2023-10-25

**Authors:** Kadejh Abdulrahman Bashekah, Moataz Emad Zagzoug, Abdulaziz Wael Banaja, Abdulaziz Abdulrahman Alghamdi, Obadah Suhail Mishiming, Mohammed Anwar Jan, Omar Akram Kemawi, Badr Ali Alharbi, Aseel Ahmed Althagafi, Sarah Mauafaq Aljifri

**Affiliations:** 1 Endocrine and Diabetes Center, Saudi Ministry of Health, Jeddah, SAU; 2 Department of Orthopaedics, King Abdulaziz Hospital, Jeddah, SAU; 3 Faculty of Medicine, King Abdulaziz University, Jeddah, SAU; 4 Faculty of Medicine, Ibn Sina National College, Jeddah, SAU

**Keywords:** knee osteoarthritis, western ontario and mcmaster universities arthritis index (womac), severity, prevalence rate, saudi arabia

## Abstract

Background

Knee osteoarthritis (OA) is a chronic and progressive knee joint condition that is influenced by multiple factors. This research aims to examine the prevalence and characteristics of knee OA among the general public in Saudi Arabia.

Methodology

This cross-sectional online survey was conducted in September 2023 in Saudi Arabia. This research used a previously developed questionnaire to validate the diagnosis of OA, which was performed in accordance with the diagnostic criteria established by the American College of Rheumatology (ACR). The Western Ontario and McMaster Universities Arthritis Index questionnaire (WOMAC) was used to examine the severity and characteristics of knee OA patients. A binary logistic regression analysis was conducted to determine the variables that influence the severity of knee OA and the likelihood of developing OA.

Results

A total of 1,019 individuals participated in this study. Around one-third of the participants (34.5%) fulfilled the ACR criteria for knee OA diagnosis. Overall, the mean WOMAC score was 34.1 (18.8) out of 96, which represents 35.5% of the maximum obtainable score and demonstrates a low degree of knee OA severity. The mean pain sub-scale score was 7.4 (3.8) out of 20, which represents 37.0% of the maximum obtainable score and demonstrates a low level of pain intensity. The mean stiffness sub-scale score was 2.7 (1.8) out of 8, which represents 33.8% of the maximum obtainable score and demonstrates a low degree of stiffness in joints. The mean physical function sub-scale score was 24.0 (14.0) out of 68, which represents 35.3% of the maximum obtainable score and demonstrates a low level of physical function difficulty. Females, older participants (above 40 years), those with high body mass index (28.8 kg/cm^2^ and higher), non-smokers, those with comorbidities, those who did not practice daily physical activity, those who had a family history of knee OA, and those who suffered from flat feet, rheumatoid arthritis, gout, lupus, or back or hip pain were more likely to develop knee OA and have severe OA (p < 0.05).

Conclusions

The findings of this study demonstrated a significant prevalence rate of knee OA and highlighted a discrepancy between the rates obtained by diagnostic criteria and those determined through clinical diagnosis. Several significant factors that contribute to the development of OA encompass lifestyle choices such as food and exercise, familial predisposition, genetic influences, and the presence of comorbidities. To effectively tackle this intricate matter, it is imperative to adopt a patient-centered strategy and prioritize early intervention.

## Introduction

Knee osteoarthritis (OA) is a chronic and progressive knee joint condition that is influenced by multiple factors. It is also the most commonly observed form of arthritis among elderly people. The condition imposes a significant financial burden on both patients and the healthcare system [1,2]. The majority of patients diagnosed with knee OA experience discomfort and a decrease in their capacity to perform regular tasks. Patient-reported outcomes play a significant role in informing clinical decision-making in situations where objective radiological measures are lacking. Consequently, the therapy of OA can be predicated on clinical factors and functional status, rather than being solely dependent on radiological observations [3,4].

The assessment of knee symptoms in individuals with knee OA holds significant importance in evaluating the progression of the illness and gauging the efficacy of therapeutic interventions. Hence, there exists a necessity for a valid and accurate metric to quantitatively evaluate the impact of OA on pain and disability in the knee joint, as well as to appraise the effectiveness of therapies [5].

Previous research on the prevalence of OA in Saudi Arabia has been undertaken but has been restricted in scope and has certain limitations. For instance, the data obtained in these studies were primarily derived from certain local regions, resulting in a small sample size [6-9]. It is worth noting that a prior investigation demonstrated that 50% of participants exhibited clinical asymptomatic conditions despite the presence of radiographic findings, and conversely, vice versa [10]. Insufficient data has been collected from a substantial sample size in Saudi Arabia about knee OA and its associated risk factors. According to the clinical guidelines, regular X-rays are not recommended for the diagnosis of OA [11]. Hence, the present study employs clinical criteria for the diagnosis of knee OA and encompasses participants from various regions within the kingdom, ensuring an adequate number of participants to establish a sample that is representative of the community. Moreover, there are variations in the prevalence of knee OA among different racial and ethnic groups [12]. Hence, it is crucial to obtain a current estimation of the prevalence of knee OA and ascertain the modifiable risk factors to facilitate timely prevention and early intervention. This research aims to examine the prevalence and characteristics of knee OA among the general public in Saudi Arabia.

The specific objectives of this study are to assess the prevalence of knee OA among the general public in Saudi Arabia, assess the characteristics of knee OA among the general public in Saudi Arabia, and identify the predictors of knee OA among the general public in Saudi Arabia.

## Materials and methods

Study design and settings

This cross-sectional online survey was conducted in September 2023 to examine the prevalence and characteristics of knee OA among the general public in Saudi Arabia.

Sampling procedure

This study sample was selected using convenience sampling, a type of non-probability sampling. This study involved participants who met our inclusion criteria and were willing to participate. On the first page of the questionnaire, participants were provided with an informed consent form and given the choice to continue participating in the study or withdraw from it. To ensure that patients comprehended the relevance of their participation, the study aims were communicated in full. In the invitation letter for the study, the inclusion criteria were outlined.

Study population and recruitment

The inclusion criteria for this study were Saudi Arabians at least 20 years old who were part of the general community. There were no limits depending on age, gender, or area of residency. Participants who did not meet the inclusion criteria were excluded. The survey link was published on social media channels (Facebook, Snapchat, WhatsApp, and Twitter) to encourage participation. The survey link was distributed through different social media pages and groups to reach participants from different regions in Saudi Arabia.

Study tool

This research used a previously developed questionnaire to validate the diagnosis of OA, which was performed in accordance with the diagnostic criteria established by the American College of Rheumatology (ACR) [9]. These criteria are outlined as follows: the initial stage was the identification of individuals who experienced knee pain for the majority of the preceding 30 days and responded affirmatively to the query, “Have you experienced pain in one or both knees in most of the preceding 30 days?” These participants were assigned a score of 1. In the second step, a score of 1 was assigned to indicate the presence of specific symptoms. These symptoms included crepitus, which refers to the experience of pain when applying pressure or compression to the knee(s). Another symptom was bony enlargement, which pertains to the perception of the knee(s) being larger than their normal size. Bony tenderness, characterized by pain when pressing or compressing the knee(s), was also considered. Additionally, the presence of morning joint stiffness lasting for a duration of 30 minutes and age exceeding 50 years were included as symptoms. A subject was diagnosed with clinical knee OA if their total score reached 3 or above, indicating fulfillment of the ACR criteria. Participants who responded negatively or affirmatively to the initial inquiry regarding knee pain experienced within the preceding 30 days and obtained a cumulative score below 3 were classified as being healthy [9].

The Western Ontario and McMaster Universities Arthritis Index questionnaire (WOMAC) was used to examine the severity and characteristics of knee OA patients. It is a self-administered composite questionnaire designed to assess pain, stiffness, and physical impairment in a three-dimensional manner. The instrument typically employed for the assessment of OA in the knee and hip is widely utilized [13]. The WOMAC Osteoarthritis Index comprises 24 items that are categorized into three distinct sub-scales, namely, pain, joint stiffness, and physical function. The pain sub-scale comprises a set of five inquiries pertaining to the experience of pain. The stiffness sub-scale comprises a total of two inquiries. The physical function sub-scale comprises a set of 17 inquiries that assess the level of difficulty experienced in performing activities of daily living. The questions in this study were evaluated using a five-point Likert scale, where each item had five response levels that corresponded to varying degrees of severity. These levels were scored from 0 to 4, with 0 indicating no intensity, 1 indicating mild intensity, 2 indicating moderate intensity, 3 indicating severe intensity, and 4 indicating extreme intensity. The scores of the items within each sub-scale were aggregated, utilizing the following ranges: pain (0-20), stiffness (0-8), and physical function (0-68). The calculation of the total WOMAC score involved the summation of the items from each of the three sub-scales, resulting in a score ranging from 0 to 96. Higher scores were indicative of poorer overall health status in individuals with OA.

The questionnaire also collected data about age, gender, body mass index (BMI), monthly income, level of education, nationality, smoking status, existence of comorbidities (such as dyslipidemia, hypertension, diabetes mellitus, and cardiovascular diseases), and occupation of the responder. In addition, the questionnaire asked about the participants’ daily physical activity level, the presence of a family history of knee OA, whether the participants were diagnosed with knee OA by their physician, and whether they had applied for sick leave because of their knee pain in the past year. Participants were asked whether they suffered from flat feet, rheumatoid disease, gout, or systemic lupus erythematosus. Moreover, they were asked whether they suffered from back or hip pain problems; whether they had pain in one or both knees for most of the past 30 days; their duration of pain; whether they felt pain when pressing on their knee/knees; whether they thought their knee/knee bones were larger than normal (hypertrophic); whether their knee/knees made a clicking or rubbing sound; whether they thought their knee/knees became stiff for the first 30 minutes in the morning; whether they had a previous knee injury due to an accident, playing a particular sport, or because of work; and whether they ate a healthy balanced diet (containing vegetables, fruits, dairy, and proteins).

Piloting of the questionnaire tool

Doctors from the Saudi Ministry of Health assessed and verified the questionnaire instrument. Participants were asked about the clarity, comprehensibility, and face validity of the questions, as well as if any were difficult to understand. In addition, participants were asked about any questions they found disrespectful or annoying. In addition, a pilot study was conducted with a small sample of the study population to evaluate their grasp of the questionnaire before its widespread adoption.

Survey translation

To promote the involvement of the general population in Saudi Arabia, the Arabic version of the questionnaire instrument was used [14].

Sample size

The minimum required sample size was 385 individuals using a 95% confidence interval, a 0.5 standard deviation (SD), and a 5% margin of error.

Ethical approval

This study was reviewed by the Institutional Review Board at the Ministry of Health, Jeddah, Saudi Arabia (approval number: KSA: H-02-J-002- A01722).

Statistical analysis

Data analysis was performed using SPSS version 27 (IBM Corp., Armonk, NY, USA). A histogram and normality metrics were used to examine the normality of the knee OA score. Based on the normality of the data, the knee OA score was presented as the mean (SD). In a binary logistic regression analysis, the mean knee OA score of the participants was utilized as the dummy variable to determine the variables that influenced the severity of knee OA, and the ACR criteria were used to identify risk factors that influenced the likelihood of developing OA. To determine statistical significance, a two-sided p-value of less than 0.05 was utilized.

## Results

Participants’ baseline characteristics

A total of 1,019 individuals participated in this study. More than half of them (57.2%) were females. Around one-quarter of the participants (24.4%) were aged 51-60 years. More than half of the participants (65.5%) were married and reported that they held bachelor’s degrees (62.8%). Around one-quarter of the participants (27.4%) reported that their family monthly income was higher than 20,000 Saudi Arabia riyal (SAR). Around 26.9% of the participants were unemployed. The vast majority (91.7%) of the participants were Saudis. Further details on the baseline characteristics of the study participants are presented in Table 1.

**Table 1:** Participants’ baseline characteristics. SAR = Saudi Arabia riyal

Variable	Frequency	Percentage
Gender		
Females	583	57.2%
Age categories		
20–23 years	83	8.1%
24–30 years	176	17.3%
31–40 years	124	12.2%
41–50 years	230	22.6%
51–60 years	249	24.4%
61 years and older	157	15.4%
Marital status		
Single	262	25.7%
Married	667	65.5%
Divorced	62	6.1%
Widowed	28	2.7%
Education		
Secondary school or lower	159	15.6%
Diploma	3	0.3%
Bachelor’s degree	640	62.8%
Higher education	217	21.3%
Family monthly income		
Less than 5,000 SAR	183	18.0%
5,001–10,000 SAR	203	19.9%
10,001–15,000 SAR	194	19.0%
15,001–20,000 SAR	160	15.7%
20,001 SAR and above	279	27.4%
Employment status		
Retired	234	23.0%
Unemployed	274	26.9%
Office work	236	23.2%
Fieldwork	84	8.2%
Both (office work and fieldwork)	191	18.7%
Nationality		
Saudi	934	91.7%

Participants’ lifestyle, health status, and prevalence of knee osteoarthritis

Table 2 presents patients’ lifestyle, health status, and prevalence of knee OA. Around one-third of the participants (34.5%) fulfilled the ACR criteria for knee OA diagnosis. The mean BMI for the study participants was 28.8 (6.2) kg/cm^2^. Around 20.5% of the participants were smokers, and 33.2% reported that they had comorbidities. Around 90.0% of the participants reported that they performed physical activity on a daily basis. Around one-quarter of the participants (24.9%) reported having suffered a previous knee injury due to an accident, playing a specific sport, or work. More than half of the participants (67.2%) reported that they ate a healthy and balanced diet. More than half of the participants (76.6%) reported that they had a family history of knee OA. Around one-third of the participants (28.7%) reported that they were diagnosed with knee OA. Around 8.6% of the participants reported that they had applied for leave during the past year due to knee pain.

**Table 2:** Patients’ lifestyle, health status, and prevalence of knee osteoarthritis.

Variable	Frequency	Percentage
Mean body mass index (kg/cm^2^) (standard deviation)	28.8 (6.2)	
Current smoker		
Yes	209	20.5%
Comorbidity history		
Yes	338	33.2%
Daily physical activity level		
Inactive	109	10.7%
Low activity that requires little effort (such as walking slowly, sitting, using the computer, light work, standing, cooking, and washing dishes)	575	56.4%
Moderate activity requiring moderate effort such as jogging, brisk walking (4 mph), heavy cleaning (window washing, sweeping, mopping), and light cycling (10–12 mph)	259	25.4%
High activity that requires high effort, such as running at a speed of 6 mph, carrying heavy loads, cycling at a higher speed (14–16 mph), playing basketball, and playing football	76	7.5%
Have you suffered a previous knee injury due to an accident, playing a specific sport, or due to work? Yes	254	24.9%
Do you eat a healthy and balanced diet (contains vegetables, fruits, dairy, and proteins)? Yes	685	67.2%
Do you have a relative (father, mother, brother, sister, uncle, aunt, uncle, aunt, grandfather, and great-grandfather) who suffers from knee osteoarthritis (knee osteoarthritis)?		
Yes	781	76.6%
Have you been diagnosed by a doctor with inflammation of the knee joints (knee osteoarthritis)?		
Yes	292	28.7%
Have you applied for leave during the past year due to knee pain you are suffering from?		
Yes	88	8.6%

Knee osteoarthritis-related risk factors

Table 3 presents the prevalence of risk factors related to knee OA. Around 13.1% of the participants reported that they suffered from flat feet. Around 5.5% of the participants reported that they suffered from rheumatoid arthritis and 0.8% suffered from lupus. More than half of the participants reported that they suffered from back or hip pain (57.3%) and felt pain in one or both knees for most of the past 30 days (57.5%). Around 38.4% of the participants reported that they had pain in both knees. More than one-third of the participants (38.6) who reported that they had knee(s) pain reported that they had been feeling pain for one to five years.

**Table 3:** Knee osteoarthritis-related risk factors.

Variable	Frequency	Percentage
Do you suffer from flat feet? Yes	133	13.1%
Do you suffer (have you been diagnosed with) rheumatoid arthritis? Yes	56	5.5%
Do you suffer (have you been diagnosed with) gout? Yes	79	7.8%
Do you suffer from (have been diagnosed with) lupus? Yes	8	0.8%
Do you suffer from back or hip pain? Yes	584	57.3%
Have you felt pain in one or both knees for most of the past 30 days? Yes	586	57.5%
In which knee do you feel pain? (n = 586)		
Right knee	201	34.3%
Left knee	160	27.3%
Both knees	225	38.4%
How long have you been feeling pain? (n = 586)		
Less than one years	204	34.8%
1–5 years	226	38.6%
6–10 years	99	16.9%
11–15 years	23	3.9%
More than 15 years	33	5.6%
Do you feel pain when pressure is applied to your knee/knees? Yes	348	34.2%
Do you think your knee/knee bones are larger than normal (hypertrophic)? Yes	83	8.1%
Does your knee/knees make a clicking or rubbing sound? Yes	404	39.6%
Do you think your knees/knees are stiff in the first 30 minutes in the morning? Yes	193	18.9%

Around 34.2% of the participants reported that they felt pain when pressure was applied to their knee(s). Around 8.1% reported that they thought their knee(s) bones were larger than normal (hypertrophic). Around 39.6% reported that their knee(s) made a clicking or rubbing sound, and 18.9% reported that they thought their knee(s) were stiff in the first 30 minutes of the morning.

WOMAC score for patients with knee osteoarthritis

Table 4 presents the mean WOMAC score for patients with knee OA. Overall, the mean WOMAC score was 34.1 (18.8) out of 96, which represents 35.5% of the maximum obtainable score and demonstrates a low degree of knee OA severity. The mean pain sub-scale score was 7.4 (3.8) out of 20, which represents 37.0% of the maximum obtainable score and demonstrates a low level of pain intensity. The mean stiffness sub-scale score was 2.7 (1.8) out of 8, which represents 33.8% of the maximum obtainable score and demonstrates a low degree of stiffness in joints. The mean physical function sub-scale score was 24.0 (14.0) out of 68, which represents 35.3% of the maximum obtainable score and demonstrates a low level of physical function difficulty.

**Table 4:** Mean WOMAC score for patients with knee osteoarthritis. WOMAC = Western Ontario and McMaster Universities Arthritis Index questionnaire

Sub-scale	Score range for the specific scale	Mean (standard deviation)	Percentage out of the maximum score for the specific scale
Pain sub-scale	0–20	7.4 (3.8)	37.0%
Stiffness sub-scale	0–8	2.7 (1.8)	33.8%
Physical function sub-scale	0–68	24.0 (14.0)	35.3%
Total score	0–96	34.1 (18.8)	35.5%

Severity profile of knee osteoarthritis

Table 5 presents the severity profile of knee OA patients. Regarding the pain intensity sub-scale, the patients reported the highest degree of severe to extremely severe problems (12.8%) when going up or down the stairs. The lowest degree of severe to extremely severe problems (3.4%) was reported when walking on flat ground. Regarding the joint stiffness sub-scale, the patients reported similar degrees of severe to extremely severe problems when they woke up in the morning (14.1%) and after sitting, lying down, or resting during the day (13.9%). Regarding the physical function difficulty sub-scale, the patients reported the highest degree of severe to extremely severe problems when they went up the stairs (14.1%), and the lowest degree of severe to extremely severe problems was when walking on flat ground (2.9%).

**Table 5:** Severity profile of knee osteoarthritis.

Number		Not any	A little	Moderate	Severe	Extremely severe
	The intensity of the pain you feel					
1	When you walk on flat ground	54.5%	26.8%	15.3%	3.1%	0.3%
2	When you go up or down the stairs	32.0%	32.8%	22.4%	9.8%	3.0%
3	At night while you are in your bed (on the bed)	58.3%	24.9%	12.4%	3.9%	0.5%
4	When sitting or lying down	52.3%	28.6%	13.3%	4.7%	1.1%
5	When standing	41.4%	32.3%	18.4%	6.0%	2.0%
	The severity of the stiffness (stiffness) you feel in your joint					
1	When you wake up in the morning	62.7%	20.5%	11.5%	4.7%	0.6%
2	After sitting, lying down, or resting during the day	53.5%	27.7%	13.2%	4.9%	0.8%
	The severity of the difficulty you are facing					
1	When you go down the stairs	51.1%	24.8%	15.0%	7.3%	1.8%
2	When you go up the stairs	37.7%	30.1%	18.1%	10.9%	3.2%
3	When stopping after sitting	45.3%	30.5%	15.3%	7.1%	1.8%
4	When standing	47.1%	28.8%	16.7%	6.0%	1.5%
5	When you bend down to the ground	50.7%	25.6%	15.8%	5.5%	2.4%
6	When you walk on flat ground	62.0%	23.0%	12.2%	2.1%	0.8%
7	When you get in or out of the car	55.3%	24.7%	14.1%	4.7%	1.1%
8	When you go shopping	49.5%	25.2%	16.6%	7.1%	1.7%
9	When you wear socks	67.6%	17.8%	10.4%	3.4%	0.8%
10	When you get out of bed	57.6%	24.5%	11.8%	5.3%	0.8%
11	When you take off your socks	70.2%	17.2%	9.3%	2.5%	0.9%
12	When you lie in bed	66.4%	20.2%	9.7%	3.3%	0.3%
13	When you get in or out of the bathtub	67.0%	20.4%	8.9%	3.1%	0.5%
14	When sitting on the chair	63.5%	23.2%	10.1%	2.7%	0.5%
15	When you sit or get up from the toilet	58.0%	26.0%	11.0%	3.5%	1.5%
16	When you do heavy housework	39.6%	28.8%	17.7%	11.1%	2.8%
17	When you do light housework	56.1%	27.4%	13.3%	2.6%	0.5%

Predictors of knee osteoarthritis and its severity

Table 6 presents the findings of binary logistic regression analysis. Females, older participants (aged above 40 years), those with high BMI (28.8 kg/cm^2^ and higher), non-smokers, those with comorbidities, those who did not practice daily physical activity, those who had a family history of knee OA, and those who suffered from flat feet, rheumatoid arthritis, gout, lupus or back or hip pain were more likely to develop knee OA and have severe OA (p < 0.05).

**Table 6:** Predictors of knee osteoarthritis and its severity. *: p < 0.05; **: p < 0.01; ***: p < 0.001. SAR = Saudi Arabia riyal

Variable	Odds ratio of developing knee osteoarthritis (95% confidence interval)	Odds ratio of having severe knee osteoarthritis (95% confidence interval)
Gender		
Females (reference group)	1.00	
Males	0.72 (0.55–0.93)*	0.43 (0.30–0.61)***
Age categories		
20–23 years (reference group)	1.00	
24–30 years	1.07 (0.44–2.57)	0.78 (0.18–3.34)
31–40 years	2.14 (0.91–5.04)	2.86 (0.78–10.46)
41–50 years	3.46 (1.58–7.59)**	5.96 (1.79–19.78)**
51–60 years	12.86 (5.95–27.80)***	10.85 (3.32–35.47)***
61 years and older	15.16 (6.83–33.63)***	12.83 (3.86–42.60)***
Marital status		
Single (reference group)	1.00	
Married	5.00 (3.36–7.42)***	6.51 (3.46–12.23)***
Divorced	5.35 (2.88–9.96)***	8.62 (3.79–19.61)***
Widowed	6.01 (2.63–13.76)***	10.81 (3.99–29.29)***
Education		
Secondary school or lower (reference group)	1.00	
Diploma	-	-
Bachelor’s degree	0.74 (0.52–1.07)	0.61 (0.40–0.93)*
Higher education	0.87 (0.57–1.33)	0.70 (0.42–1.14)
Family monthly income		
Less than 5000 SAR (reference group)	1.00	
5,001–10,000 SAR	1.02 (0.66–1.57)	0.72 (0.44–1.19)
10,001–15,000 SAR	1.36 (0.89–2.09)	0.90 (0.55–1.47)
15,001–20,000 SAR	1.51 (0.97–2.36)	0.97 (0.58–1.62)
20,001 SAR and above	1.09 (0.73–1.62)	0.48 (0.29–0.79)**
Employment status		
Retired (reference group)	1.00	
Unemployed	0.28 (0.19–0.40)***	0.39 (0.25–0.59)***
Office work	0.25 (0.17–0.36)***	0.40 (0.26–0.62)***
Fieldwork	0.20 (0.11–0.35)***	0.35 (0.18–0.68)**
Both (office work and fieldwork)	0.24 (0.16–0.36)***	0.19 (0.11–0.34)***
Body mass index category		
Lower than 28.8 kg/m^2 ^(reference group)	1.00	
28.8 kg/cm^2 ^and higher	3.10 (2.37–4.06)***	
Current smoker Yes	0.66 (0.47–0.92)*	0.36 (0.22–0.61)***
Comorbidities history Yes	3.87 (2.94–5.11)***	3.39 (2.44–4.70)***
Daily physical activity level		
Inactive (reference group)	1.00	
Low activity	0.65 (0.43–0.97)*	0.55 (0.35–0.87)*
Moderate activity	0.46 (0.29–0.72)***	0.38 (0.22–0.66)***
High activity	0.19 (0.09–0.39)***	0.13 (0.04–0.38)***
Have you suffered a previous knee injury due to an accident, playing a specific sport, or due to work? Yes	2.01 (1.51–2.69)***	1.97 (1.40–2.76)***
Do you eat a healthy, balanced diet (contains vegetables, fruits, dairy, and proteins)? Yes	1.07 (0.81–1.41)	1.31 (0.92–1.86)
Do you have a relative who suffers from knee osteoarthritis (knee osteoarthritis)? Yes	2.05 (1.30–3.26)**	0.88 (0.70–1.09)
Do you suffer from flat feet? Yes	1.55 (1.07–2.25)*	1.11 (0.70–1.76)
Do you suffer (have you been diagnosed with) rheumatoid arthritis? Yes	3.67 (2.09–6.45)***	3.73 (2.14–6.50)***
Do you suffer (have you been diagnosed with) gout? Yes	3.42 (2.13–5.50)***	2.91 (1.78–4.74)***
Do you suffer from (have been diagnosed with) lupus? Yes	13.47 (1.65–109.94)*	32.69 (4.00–267.28)**
Do you suffer from back or hip pain? Yes	3.69 (2.76–4.93)***	4.10 (2.76–6.10)***

## Discussion

Knee OA is a degenerative disorder that affects the knee joint over time and is impacted by several factors. Additionally, it is the most prevalent type of arthritis among the elderly [1]. Indeed, when assessing the progression of arthritis and the effectiveness of treatment therapies in people with knee OA, the assessment of knee symptoms is crucial [5]. Therefore, this study aimed to assess the prevalence and characteristics of knee OA among the general public in Saudi Arabia.

The findings of our study revealed that the prevalence rate of knee OA was 34.5% according to the ACR diagnostic criteria. This is higher than the percentage of patients who confirmed that they had been diagnosed by a doctor with inflammation of the knee joints (knee osteoarthritis), which was 28.7%. Interestingly, our results are similar to studies in India, where the prevalence of knee OA according to the ACR diagnostic criteria was 34.67% [15] and 28.7% diagnosed by a doctor. Knee OA prevalence was higher in another study in Saudi Arabia, which using radiographic diagnosis found that the prevalence of knee OA was 53.3% [7]. This aligns with the research conducted in Asian populations, where a prevalence of 60% or more has been identified. In contrast, the prevalence rate of knee OA in the United States, similar to our study findings, ranged between 30% and 40% [16-20]. However, the difference in prevalence rate between the ACR diagnostic criteria and the prevalence rate for those diagnosed by a doctor may be because a proportion of knee OA is asymptomatic [16], which emphasizes the role of primary care providers in the initial diagnosis and management of knee OA [17]. The significance of prioritizing a patient-centric approach lies in its emphasis on addressing the primary symptoms, diagnosis, and treatment preferences identified by individuals suffering from knee OA [18].

In our study result, a majority of the participants (67.2%) indicated that they ate a healthy and balanced diet. It is believed that there is a connection between one’s nutritional well-being and physical activity levels in relation to knee OA, implying that taking preventative measures at a younger age could potentially mitigate the development of this condition [19]. Moreover, it was found that a combination of diet and exercise leads to significant improvements in quality of life for patients with knee OA [20]. Consuming fiber-rich diets and adopting eating patterns such as the Mediterranean diet can be effective strategies for the management of OA [21]. On the other hand, the study revealed that more than half of the participants (76.6%) reported a family history of knee OA. Genetic and systemic factors may have an important role in early structural changes and pain in knee OA [22]. Moreover, BMI, muscular strength, knee pain, and the size of the medial tibial bone area appear to contribute to the genetic control and onset of knee OA [23].

This study explored the prevalence of flat feet, rheumatoid arthritis, and lupus among participants, along with the prevalence of back, hip, and knee pain. It also investigated the duration of knee pain in a subset of participants. In our study, around 13.1% of the participants reported that they suffered from flat feet. In people with knee OA, flat feet have been linked to a more significant varus angle, increased pain levels, and a greater decline in physical function [24]. Moreover, around 5.5% of the participants reported that they suffered from rheumatoid arthritis, and 0.8% suffered from lupus. More than half of the participants reported that they suffered from back or hip pain (57.3%) and felt pain in one or both knees for most of the past 30 days (57.5%). Back pain is common in individuals with knee OA and is linked to poorer clinical status indicators [25]. It has been reported that a high percentage of individuals with knee OA also report chronic back pain, with back pain often occurring before the onset of knee OA [26]. Further, around 38.4% of the participants reported that they had pain in both knees. Individuals suffering from knee OA report pain in both of their knees [27], with BMI and the extent of knee flexion identified as factors associated with knee pain [27]. However, more than one-third of the participants (38.6) who reported that they had knee(s) pain also reported that they had been feeling pain for one to five years. Knee pain was characterized as discomfort or pain in the knee that persisted for 30 consecutive days within the past three months [28]. Certain factors are associated with more severe knee pain in patients with early knee OA, including higher BMI, the presence of metabolic syndrome, hypertension, and walking limitations [29].

In this study, around 34.2% of the participants reported that they felt pain when pressure was applied to their knee(s). Knee pain in OA may be associated with increased pressure sensitivity [30], and exercise interventions can potentially alleviate this sensitivity [31]. Moreover, certain factors are related to pain severity in patients with knee OA, including the presence of a higher serum interleukin-6 level associated with pain severity in early- and advanced-stage knee OA patients [32]. Moreover, increased BMI, decrease in the degree of knee flexion, and decreased quadriceps tendon thickness are factors that increase the risk of pain in knee OA [27]. The study found that around 8.1% of the participants thought that their knee(s) bones were larger than normal (hypertrophic). In individuals with severe knee OA, there are changes in the structure of knee bones. These changes include an increase in the number of tiny bone structures called trabeculae, but these trabeculae are thinner than usual [33]. This pattern suggests that the cancellous bone, which makes up the inner part of the bone, experiences osteoporotic changes. Some participants perceived their knees as larger than normal. Patients with knee OA have higher bone mineral density and larger skeletal size [34]. Additionally, the study found that around 39.6% of the participants reported that their knee(s) made a clicking or rubbing sound. Patients who report sensations of grating, cracking, or popping sounds in or around the knee joint may have a heightened risk of developing knee OA [35]. It has been suggested that if individuals with noisy knees experience this phenomenon, they face an elevated risk of developing pain in the following year compared to those without noisy knees [35]. Indeed, the study results found that 18.9% of the participants reported that they thought their knee(s) were stiff in the first 30 minutes in the morning. Patients with knee OA exhibit decreased activity in their quadriceps muscles during everyday tasks, which might contribute to experiencing morning stiffness [36]. Moreover, it was found that prolonged walking leads to increased knee joint loading, which could contribute to stiffness as well [37].

In our study, the participants’ overall mean WOMAC score was 34.1 (out of 96), indicating a low degree of knee OA severity, accounting for 35.5% of the maximum score. Specifically, the mean pain sub-scale score was 7.4 (out of 20), representing a low level of pain intensity at 37.0% of the maximum score. The mean stiffness sub-scale score was 2.7 (out of 8), indicating a low degree of joint stiffness, amounting to 33.8% of the maximum score. Additionally, the mean physical function sub-scale score was 24.0 (out of 68), signifying a low level of physical function difficulty, constituting 35.3% of the maximum score. These findings collectively suggest that the participants generally experienced low levels of knee OA severity, pain intensity, joint stiffness, and physical function limitations. Our findings are much lower than other studies. In Morocco, the level of knee OA severity accounted for 0.91 as the total WOMAC score, and the sub-scales accounted for 0.80 for pain intensity, 0.77 for stiffness, and 0.89 for physical function limitation [38]. Other studies in Italy [39], Germany [40], and Turkey [41] showed similar results to the Moroccan study. Moreover, in early-stage knee OA, the extent of inflammation, as indicated by serum interleukin-6 levels, was linked to the severity of pain. Conversely, in advanced-stage knee OA, pain severity was associated with various alignments [32]. It was found that pain intensity in knee OA is associated with sex, age, BMI, physical activity, professional activity, marital status, and conditions of assessment [42]. Stiffening of the knee and increased muscle co-contraction have been observed in individuals with medial knee OA, potentially posing a risk to the overall joint health [43]. However, several factors contribute to diminished physical functioning in knee OA, including the intensity of knee pain, BMI, and age [44].

This study investigated several factors associated with the development and severity of knee OA, and it was found that females, older participants (above 40 years), those with high BMI (28.8 kg/cm^2^ and higher), non-smokers, those with comorbidities, those who did not practice daily physical activity, those who had a family history of knee OA, and those who suffered from flat feet, rheumatoid arthritis, gout, lupus or back or hip pain were more likely to develop knee OA and have severe OA. In fact, in a community-based elderly population, a higher prevalence of knee OA was noted among females [45]. This is associated with the structure of the cartilage in females. Vocational tasks requiring knee bending are detrimental to the structure of the cartilage in females [46]. Also, it was found that the elevated incidence of knee OA in women aged 40 and above is attributed to the fact that a significant proportion of women experience the onset of OA at a younger age [47]. In addition, it was found that a high BMI was significantly associated with knee OA [48]. Individuals with high BMI have increased diurnal strains in the articular cartilage of the knee, which may contribute to the elevated risk of OA associated with obesity [49]. On the other hand, regarding the findings about non-smokers, recent studies present conflicting evidence on the relationship between smoking and knee OA in non-smokers, where one found that high pack years of smoking were associated with slightly greater knee pain and stiffness in a cross-sectional analysis [50]. Another study found a lower prevalence of knee OA in current smokers compared to non-smokers [51]. Unlike our study findings, where non-smokers were more likely to develop knee OA. Furthermore, patients diagnosed with end-stage knee OA exhibited a range of comorbid conditions, including osteoporosis, PR sarcopenia, degenerative spine disease, diabetes, and hypertension. These comorbidities have been found to impact both physical function and overall quality of life in these patients [52]. Also, there was a substantial proportion of individuals with knee OA who did not meet public health physical activity guidelines [53]. Therefore, regular physical exercise improves functional capacity and daily living physical activity in individuals with knee OA [54]. In fact, genetic and systemic factors could play a significant role in the initial structural alterations and pain associated with knee OA. Additionally, there is evidence to suggest that BMI, muscle strength, knee pain, and the size of the medial tibial bone area may contribute to the genetic regulation and development of knee OA [22,23]. In individuals with knee OA, having flat feet was associated with a more pronounced varus angle, heightened levels of pain, and a more substantial decrease in physical function [24]. Additionally, both OA and rheumatoid arthritis patients had increased knee laxity, but the severity of laxity was associated with disease severity in OA, not in rheumatoid arthritis [55]. However, other studies did not directly address the association between rheumatoid arthritis and knee OA [56]. Some studies found no significant association between gout and radiographic knee OA [57], whereas other studies suggested a possible relationship between gout and OA, highlighting biochemical, mechanical, and immunological connections between the two conditions [2]. Lastly, in individuals with knee OA, it is common to experience back pain, which is associated with worse clinical status indicators. Additionally, a significant proportion of individuals with knee OA reported chronic back pain, often occurring before the development of knee OA [25,26].

## Conclusions

This study revealed a substantial knee OA prevalence rate, with a difference between diagnosis based on criteria and clinical diagnosis. Key factors contributing to knee OA included lifestyle (diet and exercise), family history, genetics, and comorbid conditions. To address this complex issue, a patient-centered approach and early intervention are crucial. Encouraging regular exercise and dietary improvements can enhance patients’ quality of life. Additionally, the study underscores the connection between knee OA and back pain, highlighting the importance of considering both conditions in clinical management. These findings offer valuable insights for healthcare practitioners dealing with knee OA in Saudi Arabia and beyond.

## References

[1] Hunter DJ, Bierma-Zeinstra S (2019). Osteoarthritis. Lancet.

[2] Mobasheri A, Fonseca JE, Gualillo O, Henrotin Y, Largo R, Herrero-Beaumont G, Rocha FA (2021). Editorial: inflammation and biomarkers in osteoarthritis. Front Med (Lausanne).

[3] Faucher M, Poiraudeau S, Lefevre-Colau MM, Rannou F, Fermanian J, Revel M (2002). Algo-functional assessment of knee osteoarthritis: comparison of the test-retest reliability and construct validity of the WOMAC and Lequesne indexes. Osteoarthritis Cartilage.

[4] van Baar ME, Dekker J, Lemmens JA, Oostendorp RA, Bijlsma JW (1998). Pain and disability in patients with osteoarthritis of hip or knee: the relationship with articular, kinesiological, and psychological characteristics. J Rheumatol.

[5] Bae SC, Lee HS, Yun HR, Kim TH, Yoo DH, Kim SY (2001). Cross-cultural adaptation and validation of Korean Western Ontario and McMaster Universities (WOMAC) and Lequesne osteoarthritis indices for clinical research. Osteoarthritis Cartilage.

[6] Ahlberg A, Linder B, Binhemd TA (1990). Osteoarthritis of the hip and knee in Saudi Arabia. Int Orthop.

[7] Al-Arfaj A, Al-Boukai AA (2002). Prevalence of radiographic knee osteoarthritis in Saudi Arabia. Clin Rheumatol.

[8] Al-Arfaj AS, Alballa SR, Al-Saleh SS, Al-Dalaan AM, Bahabry SA, Mousa MA, Al-Sekeit MA (2003). Knee osteoarthritis in Al-Qaseem, Saudi Arabia. Saudi Med J.

[9] Althomali OW, Amin J, Acar T (2023). Prevalence of symptomatic knee osteoarthritis in Saudi Arabia and associated modifiable and non-modifiable risk factors: a population-based cross-sectional study. Healthcare (Basel).

[10] Javaid MK, Kiran A, Guermazi A (2012). Individual magnetic resonance imaging and radiographic features of knee osteoarthritis in subjects with unilateral knee pain: the health, aging, and body composition study. Arthritis Rheum.

[11] National Institute for Health and Care Excellence (NICE) (2023). Osteoarthritis: Care and Management. https://pubmed.ncbi.nlm.nih.gov/25340227/.

[12] Zhang Y, Jordan JM (2010). Epidemiology of osteoarthritis. Clin Geriatr Med.

[13] Bellamy N, Buchanan WW, Goldsmith CH, Campbell J, Stitt LW (1988). Validation study of WOMAC: a health status instrument for measuring clinically important patient relevant outcomes to antirheumatic drug therapy in patients with osteoarthritis of the hip or knee. J Rheumatol.

[14] Abd Elrazik RK, Alfeky F, Zedan A (2022). Validity and reliability of the Arabic version of WOMAC osteoarthritis index in Egyptian patients with knee osteoarthritis. J Pharm Negat.

[15] Jadhao A, Dambhare PM (2021). Study of magnitude of knee osteoarthritis among adult population with age 40 years and above in rural area: a cross sectional study. Int J Community Med Public Health.

[16] Heidari B (2011). Knee osteoarthritis prevalence, risk factors, pathogenesis and features: part I. Caspian J Intern Med.

[17] Sasek C (2015). An update on primary care management of knee osteoarthritis. JAAPA.

[18] Tallon D, Chard J, Dieppe P (2000). Exploring the priorities of patients with osteoarthritis of the knee. Arthritis Care Res.

[19] Agha Amiri M, Doulah AH, Alivand A (2015). Nutritional status and physical activity of patients with knee osteoarthritis referred to hospitals affiliated to the Ahvaz Jundishapur University of Medical Sciences. Jundishapur J Chronic Dis Care.

[20] Mohsen Magda M, Eltomy Ekhlass M, Neima Ali R (2020). Health related quality of life following dietary and exercise interventions among women with knee osteoarthritis. Am J Nurs Res.

[21] Wei N, Dai Z (2022). The role of nutrition in osteoarthritis: a literature review. Clin Geriatr Med.

[22] Pan F (2017). Genetic and Systemic Factors in Knee Osteoarthritis and Its Symptoms. https://figshare.utas.edu.au/articles/thesis/Genetic_and_systemic_factors_in_knee_osteoarthritis_and_its_symptoms/23239625.

[23] Jones G, Ding C, Scott F, Cicuttini F (2004). Genetic mechanisms of knee osteoarthritis: a population based case-control study. Ann Rheum Dis.

[24] Gross KD, Felson DT, Niu J (2011). Association of flat feet with knee pain and cartilage damage in older adults. Arthritis Care Res (Hoboken).

[25] Wolfe F, Hawley DJ, Peloso PM, Wilson K, Anderson J (1996). Back pain in osteoarthritis of the knee. Arthritis Care Res.

[26] Burnett DR, Campbell-Kyureghyan NH, Topp RV, Quesada PM, Cerrito PB (2012). A retrospective study of the relationship between back pain and unilateral knee osteoarthritis in candidates for total knee arthroplasty. Orthop Nurs.

[27] Mermerci BB, Garip Y, Uysal RS, Doğruel H, Karabulut E, Ozoran K, Bodur H (2011). Clinic and ultrasound findings related to pain in patients with knee osteoarthritis. Clin Rheumatol.

[28] Jhun HJ, Sung NJ, Kim SY (2013). Knee pain and its severity in elderly Koreans: prevalence, risk factors and impact on quality of life. J Korean Med Sci.

[29] Alekseeva L, Taskina E, Kashevarova N (2021). AB0604 Factors associated with pain in early knee osteoarthritis (preliminary data). Ann Rheum Dis.

[30] Arendt-Nielsen L, Nie H, Laursen MB, Laursen BS, Madeleine P, Simonsen OH, Graven-Nielsen T (2010). Sensitization in patients with painful knee osteoarthritis. Pain.

[31] Baker K (2001). An update on exercise therapy for knee osteoarthritis. Nutr Clin Pract.

[32] Shimura Y, Kurosawa H, Sugawara Y (2013). The factors associated with pain severity in patients with knee osteoarthritis vary according to the radiographic disease severity: a cross-sectional study. Osteoarthritis Cartilage.

[33] Messent EA, Ward RJ, Tonkin CJ, Buckland-Wright C (2005). Cancellous bone differences between knees with early, definite and advanced joint space loss; a comparative quantitative macroradiographic study. Osteoarthritis Cartilage.

[34] Karlsson MK, Magnusson H, Cöster M, Karlsson C, Rosengren BE (2015). Patients with knee osteoarthritis have a phenotype with higher bone mass, higher fat mass, and lower lean body mass. Clin Orthop Relat Res.

[35] Wise J (2017). Noisy knees may be early sign of knee osteoarthritis. BMJ.

[36] Howe TE, Rafferty D (2009). Quadriceps activity and physical activity profiles over long durations in patients with osteoarthritis of the knee and controls. J Electromyogr Kinesiol.

[37] Gustafson JA, Anderton W, Sowa GA, Piva SR, Farrokhi S (2019). Dynamic knee joint stiffness and contralateral knee joint loading during prolonged walking in patients with unilateral knee osteoarthritis. Gait Posture.

[38] Faik A, Benbouazza K, Amine B (2008). Translation and validation of Moroccan Western Ontario and McMaster Universities (WOMAC) osteoarthritis index in knee osteoarthritis. Rheumatol Int.

[39] Salaffi F, Leardini G, Canesi B (2003). Reliability and validity of the Western Ontario and McMaster Universities (WOMAC) Osteoarthritis Index in Italian patients with osteoarthritis of the knee. Osteoarthritis Cartilage.

[40] Stucki G, Meier D, Stucki S, Michel BA, Tyndall AG, Dick W, Theiler R (1996). [Evaluation of a German version of WOMAC (Western Ontario and McMaster Universities) Arthrosis Index]. Z Rheumatol.

[41] Tüzün EH, Eker L, Aytar A, Daşkapan A, Bayramoğlu M (2005). Acceptability, reliability, validity and responsiveness of the Turkish version of WOMAC osteoarthritis index. Osteoarthritis Cartilage.

[42] Perrot S, Poiraudeau S, Kabir-Ahmadi M, Rannou F (2009). Correlates of pain intensity in men and women with hip and knee osteoarthritis. Results of a national survey: the French ARTHRIX study. Clin J Pain.

[43] Schmitt LC, Rudolph KS (2007). Influences on knee movement strategies during walking in persons with medial knee osteoarthritis. Arthritis Rheum.

[44] Sharma L, Cahue S, Song J, Hayes K, Pai YC, Dunlop D (2003). Physical functioning over three years in knee osteoarthritis: role of psychosocial, local mechanical, and neuromuscular factors. Arthritis Rheum.

[45] Koedwan C, Bi J, Teerasombut C (2016). The prevalence of knee OA in community-based elders. Thai J Public Health.

[46] Teichtahl AJ, Wluka AE, Wang Y (2010). Occupational activity is associated with knee cartilage morphology in females. Maturitas.

[47] Narasimha B, Ravish K, Ranganath TS, Sri SN (2016). A study on knee joint osteoarthritis among the women aged above 40 years, residing in the urban field practice area at tertiary care centre, Bangalore, Karnataka, India. Int J Community Med Public Health.

[48] Grotle M, Hagen KB, Natvig B, Dahl FA, Kvien TK (2008). Obesity and osteoarthritis in knee, hip and/or hand: an epidemiological study in the general population with 10 years follow-up. BMC Musculoskelet Disord.

[49] Widmyer MR, Utturkar GM, Leddy HA (2013). High body mass index is associated with increased diurnal strains in the articular cartilage of the knee. Arthritis Rheum.

[50] Dubé CE, Liu SH, Driban JB, McAlindon TE, Eaton CB, Lapane KL (2016). The relationship between smoking and knee osteoarthritis in the Osteoarthritis Initiative. Osteoarthritis Cartilage.

[51] Kang K, Shin JS, Lee J, Lee YJ, Kim MR, Park KB, Ha IH (2016). Association between direct and indirect smoking and osteoarthritis prevalence in Koreans: a cross-sectional study. BMJ Open.

[52] Kim WB, Kim BR, Kim SR (2019). Comorbidities in patients with end-stage knee OA: prevalence and effect on physical function. Arch Phys Med Rehabil.

[53] Dunlop DD, Song J, Semanik PA (2011). Objective physical activity measurement in the osteoarthritis initiative: are guidelines being met?. Arthritis Rheum.

[54] Rodrigues da Silva JM, de Rezende MU, Spada TC (2017). Educational program promoting regular physical exercise improves functional capacity and daily living physical activity in subjects with knee osteoarthritis. BMC Musculoskelet Disord.

[55] Wada M, Imura S, Baba H, Shimada S (1996). Knee laxity in patients with osteoarthritis and rheumatoid arthritis. Br J Rheumatol.

[56] Aso K, Shahtaheri SM, Hill R, Wilson D, McWilliams DF, Walsh DA (2019). Associations of symptomatic knee osteoarthritis with histopathologic features in subchondral bone. Arthritis Rheumatol.

[57] Yokose C, Chen M, Berhanu A, Pillinger MH, Krasnokutsky S (2016). Gout and osteoarthritis: associations, pathophysiology, and therapeutic implications. Curr Rheumatol Rep.

